# Computer-aided detection pragmatic threshold calibration in TB active case finding: A mixed-methods study in Tondo, Manila

**DOI:** 10.1371/journal.pgph.0006908

**Published:** 2026-08-11

**Authors:** Valentina Carnimeo, Emelie Yonally Phillips, Danica Katrina Galvan, Olivier Camelique, Mary Ruth Roxas, Marve Duka, Gina Francisco Pardilla, Maria Julieta Casillano Recidoro, Richard Hibe Castro, Farah Hossain, Helena Huerga, Catherine Hewison, Juno Min

**Affiliations:** 1 Epicentre, Paris, France; 2 Médecins Sans Frontières France, Tondo Project, Tondo, Philippines; 3 Research Office, Manila Health Department, Manila, Philippines; 4 TB Prevention and Control Section, Manila Health Department, Manila, Philippines; 5 Médecins Sans Frontières, Tokyo, Japan; 6 Médecins Sans Frontières, Paris, France; 7 Médecins Sans Frontières, Amsterdam, Netherlands; University of California Irvine, UNITED STATES OF AMERICA

## Abstract

We developed a pragmatic approach to calibrating computer-aided detection (CAD) thresholds in a tuberculosis (TB) active case finding (ACF) project in the Philippines, assessing its feasibility and acceptability in a high-burden urban setting. CAD4TB v.7 (Delft Imaging, Netherlands) software was integrated into ACF activities targeting individuals aged ≥15 years in Tondo, Manila, from November 2022. Referrals for sputum collection and Xpert testing were based on CAD scores above a threshold. We used a mixed-methods approach: 1) retrospective analysis of pre-CAD data (May-November 2022) to determine the initial threshold; 2) prospective monitoring (November 2022-October 2023) to adjust the threshold; 3) semi-structured interviews with healthcare workers and stakeholders (January-April 2023). Threshold selection combined quantitative data, user experiences, and operational observations. The initial CAD threshold of 25 matched the 35% referral rate of onsite radiologists. After two weeks, referral rose to 37.5%, prompting an increase to 28. Eight months later, referral was 32.6% with a 4.9% screening yield (Xpert positive among screened individuals). Following data and stakeholders’ input, the threshold was further increased to 32, after which referral dropped below 25% and the yield was 4.2%. Overall, 14 739 individuals were screened from October 2022 to October 2023, with a total screening yield of 4.3%, resulting in 633 TB cases. CAD threshold calibration, informed by referral rate targets, screening yield, and operational constraints can improve ACF efficiency and user confidence. Adjustments informed by real-time monitoring and stakeholder feedback helped balance sensitivity and operational feasibility in a mobile, underserved population. This framework supports tailoring CAD deployment to local epidemiology, software versioning, and system capacity. It is applicable to other high-burden settings, although further validation across diverse populations and assessment of subgroup-specific thresholds are warranted.

## Introduction

Despite being a curable illness, tuberculosis (TB) continues to be the world’s leading cause of death from a single infectious agent [1]. TB remains challenging to diagnose because of its varied clinical presentations, including asymptomatic forms, and limited access to diagnostic tools. Significant gaps persist between the estimated number of people with TB and those who are diagnosed, highlighting the critical need for innovative and effective screening and diagnostic strategies to find the missing cases.

The World Health Organization (WHO) recommends chest X-ray (CXR) as a screening and diagnostic tool for pulmonary TB [2]. However, interpreting CXR can be challenging and time-consuming for radiologists, and even more so for clinicians without specific training in X-ray interpretation with no access to radiologists. To address this challenge, new technologies have been developed such as artificial intelligence (AI)-based computer-aided detection (CAD) software, which analyses CXRs and identifies abnormalities suggestive of pulmonary TB. CAD can support non-expert readers in interpretating CXRs and may improve TB detection in high-burden settings or contexts with a shortage of specialists to interpret chest radiographs [3].

Studies evaluating the diagnostic accuracy of CAD for TB have shown performance comparable to human readers in both community-based and facility-based screening contexts [4,5]. CAD software assigns a score, between 0–1 or 0–100, based on patterns suggestive of pulmonary TB. The higher the score, the more likely the CXR is consistent with TB [6]. To implement CAD, a threshold score must be selected to match the local context, a process known as threshold calibration; individuals with a score above the threshold are referred for confirmatory testing.

As CAD software becomes increasingly available, research has revealed variability in performance across different CAD products, software versions of the same CAD product, epidemiological settings and population subgroups (e.g., people living with HIV) [7]. WHO recommends calibrating CAD thresholds based on local conditions to ensure effective TB screening and diagnosis [3]. For feasibility and acceptability, the initial threshold should reflect context-specific factors, intended use scenarios, screening objectives, and acceptable costs, and then be adjusted according to collected data. Selecting an appropriate threshold involves a trade-off between sensitivity and specificity: lower thresholds increase sensitivity but reduce specificity and raise costs due to more diagnostic testing, while higher thresholds improve specificity and reduce costs but may miss cases due to lower sensitivity [6]. Determining the appropriate threshold score for a specific context remains a crucial question.

The WHO Special Programme for Research and Training in Tropical Diseases (TDR) has published a toolkit for local calibration of CAD thresholds [7]. While this toolkit is the most scientifically rigorous approach to threshold selection, the methodology requires a comprehensive operational research study, which may require significant time, resources, and research capacity and could be impractical for some screening programmes. The Stop TB Partnership has also released a guide on CAD implementation, which outlines various calibration strategies [6–8], but lacks detailed, step-by-step instructions for their practical implementation [9,10].

Therefore, a pragmatic calibration process is needed to ensure CAD software can be effectively and efficiently implemented in high-burden, resource-limited settings, where its impact could be greatest. To address this need, Médecins Sans Frontières (MSF) and Epicentre developed and implemented a pragmatic approach to CAD threshold calibration in a TB active case finding (ACF) project in Tondo, Manila, Philippines. We describe the threshold calibration process and evaluate the feasibility and acceptability of its implementation in this context.

## Methods

### Ethics statement

The study was approved by the University of the Philippines Manila Research Ethics Board (UPMREB, Ref.: UPMREB 2022-0310-01) and by the MSF Ethics Review Board (MSF ERB, Ref. ID: 2239). Authorization to conduct the study at the implementation sites was granted by the local health authorities (Manila Health Department and National TB Program) and primary healthcare centres heads, while verbal agreement was obtained from barangay chairpersons.

The retrospective analysis of pseudo-anonymised data routinely collected prior to CAD implementation (22 May – 25 Oct 2022) and used for the initial threshold selection was exempted from ethical review by both ERBs. The study was designed and conducted in accordance with the principles of biomedical research involving human subjects of the Helsinki Declaration (2013) developed by the World Medical Association Declaration. All participants provided written informed consent; for minors, parents or guardians provided consent and minors provided written assent.

Additional information regarding the ethical, cultural, and scientific considerations specific to inclusivity in global research is included in the Supporting Information (S1 Checklist).

### Study setting and design

In 2016, a national TB survey in the Philippines revealed widespread underdiagnosis and underreporting, driven by reliance on passive case finding, low notification rates from the private sector, limited screening among high-risk groups, stigma, and insufficient healthcare resources [9]. Notably, 26% of TB cases identified in the survey were asymptomatic and therefore missed by routine services [10]. The COVID-19 pandemic further disrupted TB services. TB case notifications dropped by 37% from 419 102 cases in 2019 to 263 000 in 2020 [11]. This decline was attributed to lockdown measures, repurposing of healthcare personnel, and reduced access to diagnostic and treatment services. However, efforts to restore TB services led to a rebound in notifications raising from 229 cases per 100 000 adults in 2020 to 501 cases per 100 000 adults in 2023 [12]. The same year, the Philippines ranked fourth globally for TB incidence, accounting for 7% of the world TB cases [13].

The burden is not evenly distributed across the country. The National Capital Region (NCR), which includes Manila, consistently accounts for the largest share of nationally notified TB cases in routine Department of Health (DoH) reporting, reflecting the concentration of TB risk in densely populated, resource-limited urban settings [13]. Tondo, the largest district of Manila, exemplifies this urban vulnerability: with over 654 000 people and a density of 69 000 inhabitants/km^2^, it is one of the most densely populated areas in the world and [14] a third of its population lives in extreme poverty. These structural conditions — overcrowding, poverty, and constrained access to health services — create a high-risk environment where passive case finding is particularly likely to miss TB cases, including asymptomatic ones.

In May 2022, MSF initiated ACF activities in Tondo, District 1, as part of a comprehensive strategy for TB screening, diagnosis, treatment and prevention. The activities targeted the general population aged over 15 years and were conducted regularly (on average, three times per week), across different barangays (the smallest administrative division) within the district. ACF included CXR and symptom screening for individuals spontaneously presenting at the mobile ACF site. A mobile truck equipped with a digital X-ray machine was used to perform CXRs. The initiative was carried out with local authorities and community organizations to ensure engagement and support.

This study employed a mixed-methods approach. From May 2022 to January 2023, programmatic TB data were retrospectively analysed to support the initial CAD threshold selection. Retrospective data were accessed at two different time points: in October 2022 for data collected between May and October 2022, and in late January 2023 for data collected between November 2022 and January 2023. Between January 23 and October 23, 2023, data were collected prospectively, enabling for real-time threshold adjustment.

Semi-structured interviews were carried out from January 23 to April 14, 2023 with healthcare workers involved in the ACF project and care linkage, as well as stakeholders from MSF and the National TB Programme. In addition, direct observations of field activities were used to document the calibration process and assess its feasibility and acceptability among those involved in the project’s planning and implementation. The calibration process was guided by a framework that integrated quantitative data, qualitative insights from user experiences, operational observations and overarching screening objectives.

### CAD threshold calibration

To enhance screening capacity, CAD4TB v.7 (Delft Imaging, Netherlands) was introduced into ACF activities in October 2022.

Prior to CAD implementation, an MSF radiologist and radiographer received training at Delft headquarter, in the Netherlands, on technical installation of the equipment and use of the CAD software. During a subsequent three-week visit in Tondo, they provided on-the-job training to MSF ACF staff - including radiographers, radiologists, and clinicians - and conducted a full-day orientation for local physicians.

CAD threshold calibration followed three key steps: (1) a pre-CAD implementation phase, (2) initial threshold selection and CAD implementation, and (3) periodic threshold evaluation and adjustment based on data collected (Table 1).

**Table 1:** Timeline of CAD implementation, operational activities and threshold calibration over time.

CAD implementation phase	Pre-CAD	CAD implementation and initial threshold selection	Full integration of CAD into ACF and threshold adjustment
**Period**	May – September 2022	October 2022	November 2022 – October 2023
**Operational activities**	• Active Case Finding activities with an onsite radiologist and without CAD	• Training on CAD use • Implementation of CAD • Data collection • Data analysis for initial threshold selection • *Initial threshold selected at 25* (end of October 2022)	• Data collection • *Threshold adjusted to 28 after two weeks* (November 2022) • *Threshold adjusted to 32* (beginning of July 2023)

Initial threshold selection was based on a retrospective analysis of CXR images taken during ACF activities prior to CAD implementation (May-October 2022). These CXR images were uploaded to the CAD software, and the corresponding CAD scores were recorded. During the same pre-CAD implementation period, we also calculated the Xpert referral rate based on the onsite ACF radiologist’s interpretation. An initial CAD threshold was selected to match the CAD-based referral rate with that of the radiologists; subsequently, the threshold was adjusted twice.

Definitions used in the analysis are summarised in Table 2 and were used to evaluate threshold performance and inform iterative adjustments after initial threshold selection.

**Table 2:** Operational definitions used in the study.

Term	Definition
**Initial CAD threshold**	Selected to align the CAD-based referral rate to that of the ACF radiologists.
**Laboratory referral rate**	The laboratory referral rate is the percentage of the participants screened who have a sample collected for confirmatory test either on the basis of CAD score or symptoms.
**Maximum laboratory referral rate**	It corresponds to the maximum rate possible according to the confirmatory testing capacity within a given timeframe.
**CAD referral rate**	The CAD referral rate is the percentage of the participants screened who have a sample collected for confirmatory testing only on the basis of CAD score (excludes those with CAD score below threshold who are referred for confirmatory testing due to either symptoms or radiologist interpretation).
**Maximum CAD referral rate**	It corresponds to the maximum rate that is possible according to the confirmatory testing capacity within a given timeframe.
**CAD sample collection rate**	The proportion of samples collected among participants referred for sample collection based on CAD score greater than threshold (% of *CAD-referred participants* who had a sample collected).
**Xpert tested rate**	The proportion of samples tested by Xpert among samples collected.
**Xpert positivity rate above threshold**	The proportion of samples from participants with CAD score above threshold tested with Xpert who had a positive result for TB (does not include repeat tests when first test was inconclusive – trace, inconclusive).
**Screening yield**	The proportion of Xpert positive among all screened participants (irrespective of reason for referral for sample collection, so includes participants referred on the basis of symptoms or radiologist interpretation who have a CAD score below threshold).

### Screening algorithm and follow-up procedures

**Fig 1:**
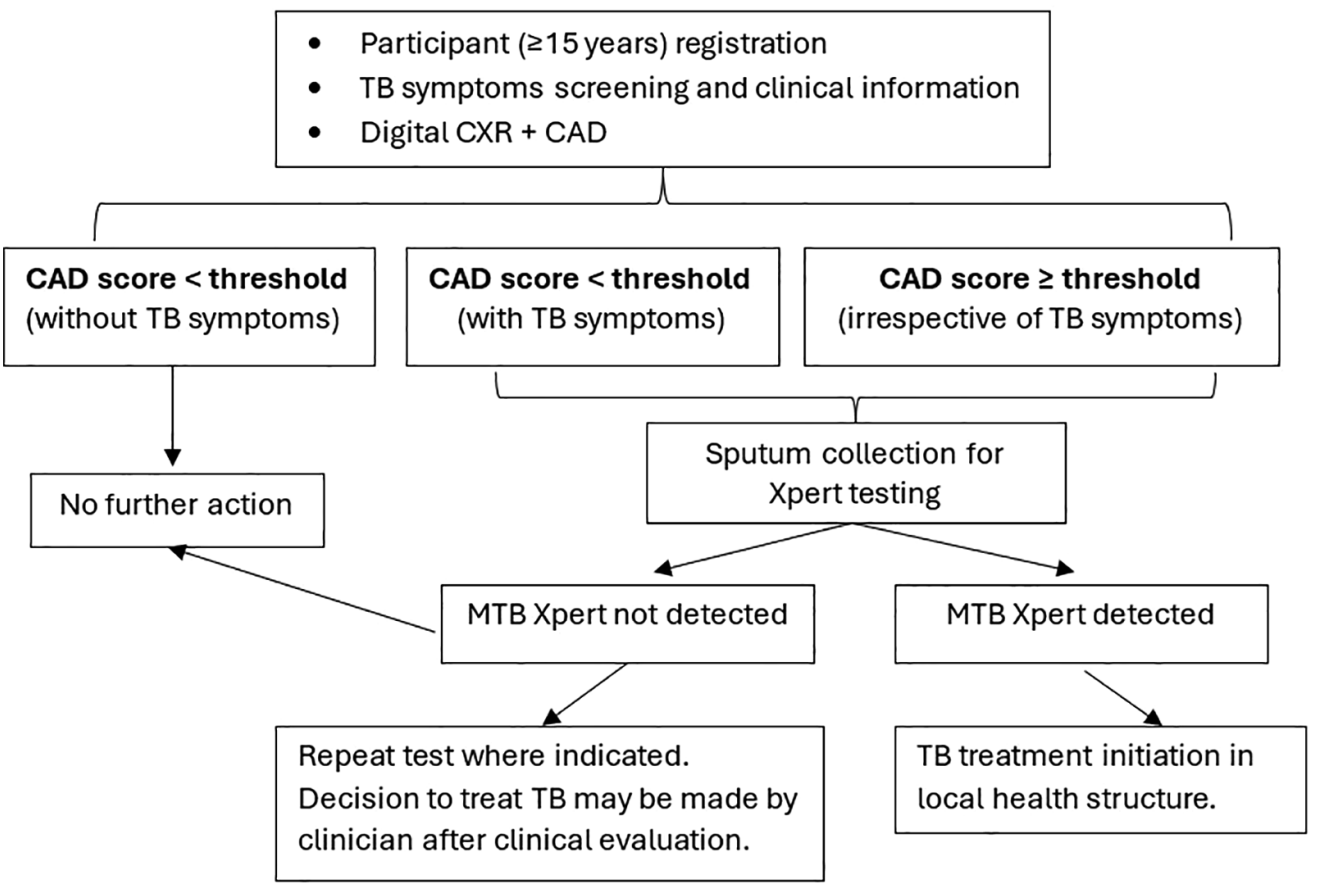
Screening and diagnostic algorithm used during the ACF intervention in District 1, Tondo, among people aged 15 years or older.

All participants underwent TB symptom screening and digital CXR analysed by CAD. Individuals already on TB treatment were excluded from CXR screening. Onsite sputum collection was triggered either by a CAD score above threshold or a positive symptom screening result regardless of the CAD score (Fig 1).

Symptom screening was considered positive if participants reported any of the following: cough ≥2 weeks, haemoptysis, fever, weight loss, or night sweats. Sputum specimens were sent daily to Manila Health Department (MHD) laboratories for Xpert testing (Xpert MTB/RIF Ultra [Cepheid, Sunnyvale, USA]). Results were communicated to health centres and patients with positive Xpert results (confirmed TB cases) were contacted for follow-up. Confirmed TB cases were followed up by an MSF team that conducted household contact tracing and investigation in line with National TB Programme guidelines.

### Data collection and analysis

Quantitative data was collected using paper-based case reporting forms and then entered pseudo-anonymised into a secure, password-protected database (REDCap Software). Manual and automatic/real-time consistency checks were performed to ensure data quality. Data manipulation and analyses were performed using R software and Stata version 16 (College Station, Texas, USA).

Descriptive analyses included counts with proportions. Sensitivity of CAD at the different given thresholds was evaluated by calculating receiver operating characteristic (ROC) curve.

Semi-structured interviews were conducted with all healthcare workers trained on CAD and using the software for ACF activities (users), involved in results interpretation, and/or making treatment decisions based on CAD score (n = 10). Interviews with healthcare workers explored user knowledge and experiences of digital X-ray and CAD software, project adaptations with the implementation of CAD, and necessary facilitating conditions for including CAD in an ACF activity. Stakeholder interviews (n = 5) were conducted with staff from the MSF Philippines project and the National TB Programme to explore the planning and implementation of the ACF project using CAD, challenges and adaptations that emerged throughout the project, broader contextual, environmental and policy level factors that shaped the project, and considerations and lessons learned for project replication. Four ACF site observations of one hour duration were conducted to document two different ACF site conditions, patient flow, interactions, and environment. All interviews were audio-recorded and transcribed. Using the Nvivo software, thematic analysis was conducted on all transcriptions, interview notes, and observation sheets.

## Results

### 1. Pre-CAD implementation phase

During the five months of ACF conducted in the pre-CAD implementation phase (May to October 2022), onsite radiologists referred approximately 35% (224/641) of participants for Xpert testing based on CXR findings suggestive of TB. To enhance CAD acceptability and maintain workflow continuity, an initial CAD threshold was selected to match the radiologists’ referral rate. It was hypothesized that aligning the screening sensitivity of CAD with that of the ACF radiologists would facilitate its integration into existing workflows and would be more acceptable to patients, medical staff (including the radiologists, radiographers and clinicians) and the entire ACF team, as it would minimize disruption of the established practices.

### 2. Initial threshold selection and CAD implementation phase

For initial threshold selection, a total of 641 CXRs from the pre-CAD implementation phase (digital imaging and communications in medicine [DICOM] format,) were uploaded to the CAD4TB software and the CAD scores recorded and ranked sequentially from highest to lowest. The threshold score that would refer 35% (224/641 = 35%) of individuals for Xpert testing was identified as the 224^th^ highest score (27.03, Table 3).

**Table 3:** Sequential list of CAD scores for pre-CAD CXRs.

Number	CAD score	Xpert Referral rate
220	27.37	34.3%
221	27.31	34.5%
222	27.20	34.6%
223	27·06	34.8%
**224**	**27.03**	**35.0%**
225	26.88	35.1%
226	26.73	35.3%
227	26.66	35.4%
228	26.51	35.6%

However, a slightly lower initial threshold of 25 was selected to maximize sensitivity at the start, with the intention of adjusting upwards as needed. The laboratory testing capacity was a critical constraint. Real-time assessment of laboratory capacity was done in collaboration with Manila Health Department (MHD) and considered the number of GeneXpert machines and modules, medical technician capacity, and capacity for re-routing overflow when capacity was exceeded. Interviews with MHD and MSF ACF staff also indicated the importance of maintaining low turn-around time (TAT) for results to meet the needs of the highly mobile population of Tondo (Table 6). Taking into account the laboratory testing capacity, the maximum number of referrals by CAD for sputum collection was 30 per day, while the ACF site screened up to 87 individuals per day. Therefore, the maximum CAD referral rate was 35% (30/87), which was important to record because the CAD referral rate could not exceed this figure without increasing testing capacity or impacting TAT of the results. At an initial threshold of 25, the CAD referral rate was expected to meet or even exceed this limit, necessitating close monitoring. To expand laboratory testing capacity, an additional GeneXpert machine was purchased, along with recruitment of an extra MSF laboratory technician. Another GeneXpert machine was later purchased with hiring of an additional technician.

### 3. Threshold evaluation and adjustment phase

Throughout the process, threshold adjustments were guided by screening objectives, testing capacity, and user feedback. Monitoring data on CAD referral rates and Xpert positivity rates above threshold- in addition to consultations with users, informed threshold evaluation and adjustment, ensuring alignment with operational realities and testing capacity constraints while maintaining the highest possible sensitivity and user acceptability. Threshold evaluations were conducted frequently in the early stages of implementation and less often over time as confidence in CAD performance grew. The first threshold evaluation occurred two weeks after full integration of CAD into ACF. At the initial threshold of 25, CAD referral rate was 37.5%, exceeding the maximum CAD referral rate of 35%. Retrospective analysis showed that the CAD referral rate at a threshold of 28 would have been 32.9% (29/88), aligning with operational limits. The threshold was therefore increased to 28.

Over the following months, a threshold of 28 consistently yielded a CAD referral rate of 32–34%, which was feasible and below the maximum CAD referral rate. After nearly eight months, with a CAD referral rate of 32.6% and an Xpert positivity above threshold of 15.2% (Table 4), a more nuanced evaluation was conducted.

**Table 4:** Screened patients, CAD referral rates for Xpert, Xpert tested rate, Xpert positivity rates by thresholds during the full implementation and CAD threshold adjustment phase.

CAD threshold	25	28	32	Total
**Screened patients (N)**	88	7 994	5 658	14 739
**Patients referred for Xpert test according to the CAD threshold** **(N, %)**	33 (37.5)	2 608 (32.6)	1 396 (24.7)	4 078 (27.7)
**Xpert tested** **(N, %)**	32 (97.0)	2 585 (99.1)	1 377 (98.6)	3 994 (97.9)
**Xpert positivity (n/N, %)**	2/32 (6.2)	392/2 585 (15.2)	239/1 377 (17.4)	633/3 994 (15.8)
**Screening Yield** **(Xpert positivity among screened patients) (%)**	**2.3**	**4.9**	**4.2**	**4.3**

Xpert positivity rates were analysed by CAD score intervals overall and near the threshold. While scores 28–31 had an Xpert positivity rate of 1.5%, scores 32–35 had a rate of 3.5% (Table 5). Because increasing the threshold to 32 would result in more efficient use of Xpert testing while only leaving a few TB cases undetected, the decision was made to increase the threshold to 32.

**Table 5:** Xpert positivity rates by CAD score intervals near the threshold of interest (24-39) before adjusting to a threshold of 32.

CAD score	Total sputum (N)	Xpert positive (n)	Xpert positivity (%, n/N)
36–39	317	10	3.2
32–35	347	12	3.5
28–31	473	7	1.5
24–27	53	1	1.9

**Fig 2:**
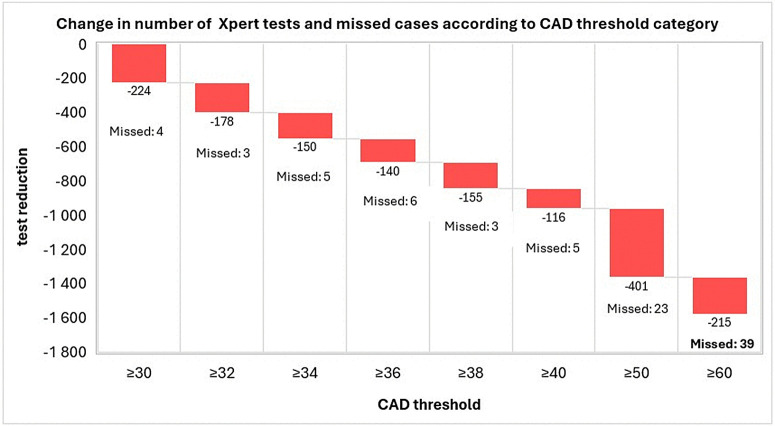
Potential missed TB cases according to CAD threshold.

From July to October 2023, a threshold of 32 resulted in a CAD referral rate of 24.7%, an Xpert positivity above threshold of 17.4%, and a screening yield of 4.2% (Table 4). The evolution of Xpert positivity rate above threshold according to CAD threshold is illustrated in Fig 2. This adjustment from 28 to 32 saved 402 (= 2 218 - 1 816) Xpert tests while hypothetically missing only seven TB cases (2% of 340 total cases diagnosed; Table 6).

**Table 6:** Trade-offs between case detection, diagnostic workload, and missed tuberculosis cases at different CAD thresholds.

CAD threshold	Screened individuals CAD ≥ threshold	Sputum collected	Xpert positive above threshold	N. of additional tests done in relation to previous CAD score category	Missed confirmed TB cases compared to the previous CAD score category
	(N)	(N)	(N, %)	(N)	(N, %)
≥28	2 238	2 218	340 (15.3)		
≥30	2 013	1 994	336 (16.9)	224	4 (1.2)
≥32	1 833	1 816	333 (18.3)	178	3 (0.9)
≥34	1 679	1 666	328 (19.7)	150	5 (1.5)
≥36	1 538	1 526	322 (21.1)	140	6 (1.9)
≥38	1 391	1 371	319 (23.3)	155	3 (0.9)
≥40	1 265	1 255	314 (25.0)	116	5 (1.6)
≥50	860	854	29 (34.7)	401	23 (7.9)
≥60	643	639	252 (39.4)	215	39 (15.5)

**Fig 3:**
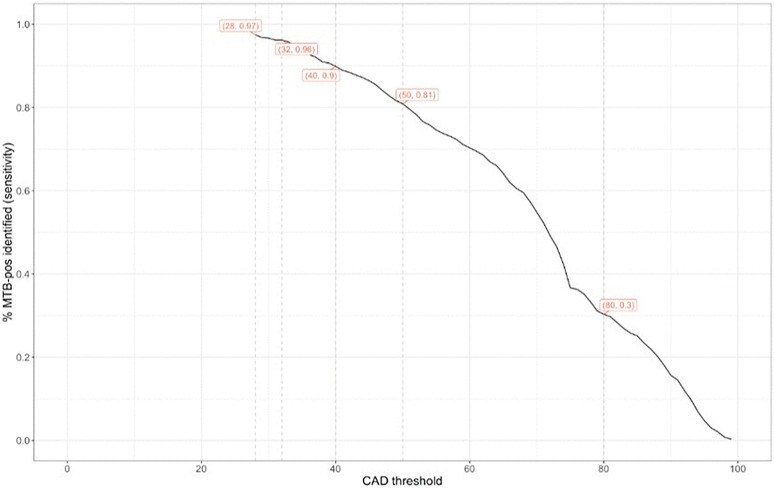
Sensitivity of CAD at different threshold.

In contrast, the manufacturer-recommended threshold of 60 (for version 7 of CAD4TB) would have saved 1 579 tests but missed 88 cases (25.9%), which was deemed unacceptable. Missed cases grow significantly at higher thresholds, especially ≥50 and ≥60 (Fig 2). Using thresholds of 28 and 32 allowed for a high level of sensitivity amongst those with a CAD score above threshold (% of Xpert MTB-positive individuals correctly classified by CAD), 96% and 97% respectively (Fig 3).

### User feedback and acceptability

Interviews with users highlighted tensions in confidence in CAD performance. While users were aware that CAD can perform as well as human readers, according to the recent literature, case-by-case discrepancies between the CAD output and the interpretation by the radiologist or TB doctor negatively impacted user confidence (Table 6). Perceived false positives were noted as wasting time and resources, while perceived false negatives were described as missing a person with TB. However, users noted that, within the context of TB screening, missing some people with TB was unavoidable regardless of screening method and ultimately acceptable as these individuals would likely be identified later if their TB disease progressed (Table 7).

**Table 7:** Qualitative evidence for CAD threshold calibration.

Theme	Finding	Representative Quote
**Feasibility of CAD threshold**	Laboratory capacity was limited by number of GeneXpert machines, number of working modules, and limited human resources	*Our machines are only four modules. And we cater six districts of Manila with 44 health centers confirmatory... And also... we have only 4 med techs in District 1 and we cater to 10 health centers. – Manila Health Department Stakeholder*
	Increased TAT would risk increasing loss to follow up in the highly mobile population in Tondo	*You need to be sure that you have Xpert machine available, that you have a short turn-around time to get the result because otherwise people will just move. Now, here we have a mobile population some in some places and people so just they just moved to another place because sometimes they’re here just because they work in the port for example. And so, the short-turn-around time for the results are important. – MSF ACF Staff*
	Threshold needs to remain flexible to maintain feasibility in changing conditions	*I’m not sure if the current threshold is working with the numbers that we’re finally pushing with the ACF... I don’t know if we’re overloading Xpert versus before when we had less patients. So that could have impacted flow. – MSF ACF Staff*
**Acceptability of CAD threshold performance**	Discrepancies in interpretation negatively impact confidence in CAD threshold performance	*On a scale of 1–100, I have a confidence of 70. Because there are some (cases) that are negative, but we can clearly see that there is something there and it should be not negative. - MSF ACF Staff*
		*[We are] lucky to have the CAD with the benefit of the radiologist who can look at it and then maybe disagree, override it, and then refer it anyway. Because the end goal is still just like to do justice to the patient, kind of never miss patients who need medical care. –MSF ACF Staff*
	Missing some patients in screening is unavoidable with any method	*It’s very OK that right now - test’s negative, mild symptoms - we don’t take any action. Because eventually, if it’s TB, the symptom persists. And so, I think... this window of observation, clinically speaking, this is something very normal to do. – MSF ACF Staff*
	Balancing sensitivity and specificity is essential to meeting project aims	*There’s a sweet spot for the threshold, so they, they just need to find it out. So that, you know the level of sensitivity and specificity… If they can find that sweet spot, it will be very, the CAD box will be very, very good. – MSF ACF Staff*
		*I think there’s only one thing that I would like to, you know, not change, but the power… to have the patient tested. When we see a…. the below threshold ones. We would like to have it tested so it can be confirmed. Yeah, but it uses resources, so maybe not. – MSF ACF Staff*
	Data-driven threshold adjustments improve user control and confidence in CAD performance	*I think the current setup is doing OK. But let’s see if ever there’s gonna be more discrepancies that will be encountered. Then maybe we should adjust the threshold. It depends on the data that we’re getting. - MSF ACF Staff*

The tension in user expectations for CAD threshold performance was further compounded by the acknowledgment of feasibility constraints in terms of laboratory capacity and TAT. Users described a ‘sweet spot’ for the CAD threshold, that balanced sensitivity with a feasible referral rate, noting that referring too many samples would not only overwhelm the laboratory but impact loss to follow up if TATs became too long (Table 7). Users also noted that this ‘sweet spot’ was flexible and dependent on the number of working Xpert modules and the number of people accessing the ACF site.

Interviews with users also highlighted the growing confidence in CAD performance based on training and experience, collected performance indicators, and confidence in the pragmatic calibration process to align CAD thresholds with project goals (Table 7). Increased experience with and understanding of CAD performance, including limitations, improved staff acceptance as they felt more confident in being able to adjust the project and their work to use CAD effectively and compensate for limitations. Additionally, users frequently referenced the project data showing performance indicators, which was linked to growing confidence that the selected threshold was performing in line with screening objectives. Furthermore, the pragmatic calibration approach improved user confidence by ensuring that threshold selection and adjustment was data-driven and flexible (Table 7).

## Discussion

The integration of CAD into ACF activities was methodical and phased, providing both a detailed guidance and a case study for adaptable threshold calibration in a complex setting. Using a pragmatic calibration method, the screening yield was 4.3% for the entire analysed period (ranging from 2.3% with a threshold of 25 up to 4.9% with a threshold set of 28), and a total of 633 TB cases were detected, while a manufacturer-recommended threshold of 60 would have saved tests but missed 15.5% of cases. This outcome highlights the importance of CAD threshold calibration in maximizing screening yield while maintaining operational feasibility.

As guidance is limited, we developed our own pragmatic calibration approach, which integrated aspects of feasibility and acceptability. Rather than relying on fixed thresholds or external recommendations, our calibration strategy was grounded in local realities—epidemiological, logistical, and social.

CAD threshold calibration must be adapted to the context in which it is implemented to ensure an appropriate threshold is selected to balance sensitivity and specificity and to match local priorities and needs. In the case of ACF in Tondo, CAD was implemented in a densely populated, high TB burden setting with a very mobile population. A recent study by Kagujje et al. (2023) demonstrated that the performance of CAD systems, including CAD4TB v7, decreased among people previously treated for TB because of changes in lung shape and texture [14]. Other studies of CAD systems have also highlighted that the sensitivity and specificity at a set threshold may differ according to factors such as age, gender, and history of previous TB [5,9,15–17]. This variability underscores the need to ensure that the selected threshold is appropriate to the local context. It also highlights the need to monitor the performance of CAD thresholds over time, disaggregated by relevant subgroups, and to consider setting differential thresholds for different subgroups. Another point to consider in threshold selection is the difference in performance of CAD software versions. Before selecting the initial threshold, we consulted CAD experts, who advised that CAD4TB version 7 would likely require a lower threshold than version 6 for screening purposes. This insight is also supported by Fehr and colleagues [18] who emphasize the risks of using the same threshold across different software versions without accounting for performance variations and highlight the critical need for transparency and clear guidance when transitioning between versions of CAD software. This suggests the potential need for threshold adjustment after upgrading to new software versions.

Assessment of the local healthcare system and the ability to integrate CAD-supported TB screening activities into the health system are also critical for establishing a feasible CAD threshold. Collaborating with the Manila Health Department allowed for discussions on logistical considerations for feasibility, such as laboratory capacity, potential rerouting options if capacity was exceeded, and managing sputum sample flow to avoid backlogs. To align with these operational constraints, a simple calculation was used to determine the maximum number of participants that could be referred by CAD for sputum collection per day. This maximum CAD referral rate should never be exceeded and defines the lower limit of the threshold score (i.e., a threshold below this lower limit will refer too many individuals for testing and overwhelm testing capacity).

Systematic monitoring of CAD referral rates and Xpert positivity rates above threshold facilitates threshold adjustments to keep in line with project targets and objectives. Tracking these indicators also allows for monitoring feasibility and provides data points that can build confidence in CAD performance. In Tondo, the initial threshold score of 25 aimed to match the local radiologists’ referral rate and provided the highest sensitivity deemed feasible in the local context. However, during the 2 weeks after CAD implementation, the CAD referral rate exceeded the maximum laboratory capacity. Although ACF staff were initially resistant to reducing the sensitivity of screening, reviewing the GeneXpert positivity rates disaggregated by CAD score revealed that increasing the threshold to reach a more feasible CAD referral rate resulted in minimal missed cases and was therefore deemed an acceptable trade-off for improved specificity and testing efficiency. Based on CAD referral data and the maximum CAD referral rate, the threshold was raised to 28. Ongoing monitoring of CAD referral rates, Xpert positivity rates above threshold and operational feasibility and acceptability allowed for continued threshold evaluation and after 8 months, the threshold was increased again to 32. This adjustment saved 402 Xpert tests while likely missing very few cases, aligning with screening objectives and increasing user acceptance. Based on interviews with users, the growing acceptance appeared to be a result of increased comfort working with the CAD software and outputs, increased confidence in CAD due to the performance monitoring, and discussions about the role of CAD and screening in the context of the evolving project design. David et al. (2023) similarly found that reinserting user control in the process through the possibility of ‘tweaking the algorithm’ by adjusting the threshold allayed some concerns and improved user confidence in CAD performance [19,20].

Threshold calibration also carries direct economic implications: a higher threshold lowers per-screening costs and reduces pressure on laboratory capacity, but risks missing cases whose delayed diagnosis generates downstream costs for both the health system and affected individuals. Dedicated cost-effectiveness analyses of CAD threshold calibration in different programmatic contexts are needed to further characterise these trade-offs.

While our CAD pragmatic calibration method offers a replicable framework, the limitations of our study must be acknowledged. First, the calibration process was conducted in a high-burden urban setting, which may limit generalizability of our findings to rural or lower-burden contexts. Second, we did not have access to reliable data for HIV co-infection and other comorbidities such as diabetes, which may play a future role in threshold calibration. The lack of this information limited our ability to explore tailored threshold selection approaches for subgroups. Finally, our study evaluated a calibration approach only with CAD4TB version 7 and while this should be generalizable to other CAD products and versions, this is an area that may need more experience to validate.

## Conclusions

The implementation of CAD in Tondo’s ACF activities illustrates the value of a carefully phased and context-sensitive approach to TB screening with CAD. We selected an initial threshold and adjusted it based on data and key indicators, while considering practical constraints and population characteristics to keep screening effective and manageable. Importantly, the calibration process was not static: it evolved through continuous monitoring and stakeholder feedback, ensuring that the system remained responsive to changing needs and constraints. These findings reinforce the importance of tailoring CAD deployment to local contexts, including software versioning, population mobility, and health system capacity. As digital tools continue to have a more central role in public health strategies, flexible approaches like the one described here can help projects maximize their impact without overstretching resources.

Future work should aim to validate this pragmatic calibration approach across diverse settings and populations, including various subgroups, rural areas and those with different TB epidemiology. Expanding data collection to capture subgroup-specific performance will enable more tailored threshold adjustments, helping to improve effectiveness and efficiency in screening outcomes. Longitudinal studies are needed to evaluate the impact of threshold calibration on TB case detection, overall patient care and transmission of disease, including the cost-effectiveness of threshold selection and adjustment. As CAD technologies evolve, transparent performance reporting across CAD solutions and software versions as well as clear global guidance on calibration practices will be essential. Ultimately, the goal is to develop adaptable, evidence-based calibration models that can be integrated into national TB programs and scaled across heterogeneous health systems.

## Supporting information

S1 Checklist Inclusivity in global research. (DOCX)
