# Supplementary material for: Computer-aided detection pragmatic threshold calibration in TB active case finding: A mixed-methods study in Tondo, Manila
Source: PLOS Glob Public Health. 2026 Aug 11;6(8):e0006908. doi: 10.1371/journal.pgph.0006908 (PMC13460636; doi:10.1371/journal.pgph.0006908)
Supplement: Supplementary file 1 [file pgph.0006908.s001.docx]

Inclusivity in global research

PLOS’ policy on inclusivity in global research aims to improve transparency in the reporting of research performed outside of researchers’ own country or community and ensures that PLOS publications reporting global research adhere to high standards for research ethics and authorship. Authors of relevant research articles may be asked to complete the questionnaire below, which outlines ethical, cultural, and scientific considerations specific to inclusivity in global research. This questionnaire may be requested when researchers have travelled to a different country to conduct research, if research uses samples collected in another country, research with Indigenous populations or their lands, or if research is on cultural artefacts. Researchers travelling to another country solely to use laboratory equipment will not normally be required to complete the questionnaire. However, the questionnaire can be requested at the journal’s discretion for any submission – if you have been requested to complete this questionnaire by the PLOS journal you submitted to, please do so.

Please complete the questionnaire below and include this as a Supporting Information file with your manuscript. Note that if your paper is accepted for publication, this checklist will be published with your article in the supporting information files. Please ensure that you reference the checklist in the main body of your manuscript. We suggest adding a subsection ‘Inclusivity in global research’ to your Methods section and adding the following sentence: “Additional information regarding the ethical, cultural, and scientific considerations specific to inclusivity in global research is included in the Supporting Information (S1 Checklist)”

The questions have been designed to be applicable to a wide range of study types, and there are subsections for both human subjects research and non-human subjects research. If any of the questions are not relevant to your research please mark them as “N/A” as appropriate.

**Ethical considerations, permits and authorship**

*This section is applicable to all research types.*

Provide details as to who granted permissions and/or consent for the study to take place in the Methods section of your manuscript. This should include the names of **all** ethics boards, governmental organizations, community leaders or other bodies that provided approval for the study. If individuals provided approval refer to these people by their role or title but do not list their name(s).

Reported on page number: Page 8, lines 199-202

If there were any deviations from the study protocol after approval was obtained please provide details of these changes in the Methods section of your manuscript.

Reported on page number: N/A

Did this study involve local collaborators that are residents of the country where the research was conducted or members of the community studied? If you do not have any authors from said communities, please provide an explanation for this below.

Yes, the study involved local collaborators from the country where the research was conducted. A local study coordinator and the ACF (Active Case Finding) manager, who is a local medical doctor, were involved in study implementation and data collection. They are both co-authors.

In addition, local researchers contributed to the qualitative component of the broader study. This component is primarily described in a separate publication, in which these local collaborators are included as co-authors. In the present manuscript, the qualitative findings are only briefly referenced, and the related activities were coordinated by EYP. For this reason, the local collaborators involved in the qualitative work are not included as co-authors in this specific article. Authorship for this manuscript reflects contributions specific to this analysis.

Everyone listed as an author should meet PLOS’ criteria for authorship and all individuals who meet these criteria should be included in the author byline, rather than the acknowledgements. For further information please see the journal’s Authorship Policy.

**Human subjects research (e.g. health research, medical research, cross-cultural psychology)**

Did you obtain written informed consent from a representative of the local community or region before the research took place? How did you establish who speaks for the community? Details of written informed consent obtained from study participants should be reported separately in the Methods section of your manuscript.

Yes, written permission to engage with the community was obtained from local leadership prior to the start of the study. In the Philippines, barangay leaders are democratically elected representatives of the community and serve as the recognized authority at the local level. We therefore coordinated with and obtained approval from the incumbent barangay chairperson, who is mandated to represent the community and facilitate external activities within the barangay.

Details of written informed consent obtained from individual study participants are provided separately in the Methods section, lines 207-208.

How did members of the local community provide input on the aims of the research investigation, its methodology, and its anticipated outcome(s)?

We engaged with a local researcher early in the development of the study protocol through a dedicated consultation call. This discussion provided important contextual insights that informed the study aims and design. In addition, local colleagues contributed substantially by reviewing the proposed research questions, assessing the feasibility of the study procedures, and suggesting appropriate messaging. Their input helped ensure that the investigation was contextually relevant, methodologically appropriate, and aligned with anticipated outcomes that are meaningful to the local community.

When engaging with the local community, how did you ensure that the informed consent documents and other materials could be understood by local stakeholders?

Informed consent documents and study materials were translated into the local language to ensure accessibility. We used clear, culturally appropriate, and non-technical language to facilitate understanding among local stakeholders. Additionally, local collaborators reviewed these materials to ensure clarity, relevance, and suitability for the target population.

Will the findings of the research be made available in an understandable format to stakeholders in the community where the study was conducted (e.g. via a presentation, summary report, copies of publications, etc.)? Please provide details of how this will be achieved.

The findings of this research have been shared with key stakeholders, with broader community dissemination occurring both indirectly and, more recently, through a formal event.

Study results were formally communicated to local health authorities through meetings and the sharing of study reports and presentation materials, ensuring that findings could inform policy and implementation decisions related to CAD use and calibration.

At the community level, information about the implementation and use of CAD was conveyed through the routine activities of community health workers. During their interactions with community members, they provided explanations about the purpose and use of the intervention, contributing to general awareness and understanding.

In June 2026, a formal dissemination event is organised in Manila, bringing together all stakeholders including community members, to share the results of this study alongside those of a related study.

**Non-human subjects research using specimens/ animals collected as part of the study, or those housed in archival collections. Examples include archaeology, paleontology, botany and zoology.**

Did the permission you obtained from a local authority to perform the study include an agreement on access to outputs and benefit sharing? This may include procedures to enable fair distribution of the benefits and resources arising from the research performed. Please include any details of Prior Informed Consent and Benefit Sharing Agreements obtained. These may be required by field-specific regulations, for example the Convention on Biological Diversity (CBD) and the associated Nagoya Protocol.

N/A

If the material used in your study was imported, please A) provide the year it was imported and B) indicate whether permits were obtained to import/export the materials used, C) provide details of any permits obtained. If this information is not available, please indicate this.

N/A

If you used archival specimens, please state how the material used in your study was acquired by the institute it is held in and provide details of any permits obtained for the original excavations/ sample collection. If this information is not available, please indicate this.

N/A

How was the potential cultural significance of the materials collected in your study to local communities considered in your research design? Were Indigenous peoples and/or local researchers and institutions involved with archaeological excavations / collection of specimens? If so, please provide a description of their involvement.

N/A

If your manuscript includes photographs of human remains please indicate whether authors obtained permission from descendants or affiliated cultural communities to do so.

N/A
