# Supplementary material for: Computer-aided detection pragmatic threshold calibration in TB active case finding: A mixed-methods study in Tondo, Manila
Source: PLOS Glob Public Health. 2026 Aug 11;6(8):e0006908. doi: 10.1371/journal.pgph.0006908 (PMC13460636; doi:10.1371/journal.pgph.0006908)
Supplement: Supplementary file 2 [file pgph.0006908.s003.docx]

Thank for your consideration of our manuscript " Computer-Aided Detection Pragmatic Threshold Calibration in TB Active Case Finding: A Mixed-Methods Study in Tondo, Manila." (Manuscript N° PGPH-D-26-00748) for publication in PLOS Global Public Health. We sincerely thank the reviewers for their thoughtful comments. We hope to have addressed these in the responses provided below and in the revised manuscript.

| **Editor (E)** | **Comments** | **Responses to Reviewers** |
| --- | --- | --- |
| E.1 | Please include a complete copy of PLOS’ questionnaire on inclusivity in global research in your revised manuscript. | We filled the form and made modifications into the article accordingly. (line 199-202, 209-210). Attached the completed form. |
| E.2 | Code sharing for submissions in which author-generated code underpins the findings in the manuscript. In these cases, all author-generated code must be made available without restrictions upon publication of the work. | We appreciate PLOS's commitment to open science and reproducibility. Regarding the request for code sharing, we respectfully submit the following explanation. The statistical analyses underpinning this manuscript were conducted using Stata and the analytic scripts are structurally linked to the restricted dataset described in our data availability statement. Because the dataset cannot be made publicly available for ethical and regulatory reasons (as detailed in the response to point R1) the scripts themselves cannot be shared in a meaningful or responsible way: they reference internal file paths, variable naming conventions, and data structures that are inseparable from the restricted source data. Sharing the code in isolation would therefore neither enable nor approximate reproducibility. We are committed to transparency and are happy to provide a detailed description of all statistical methods in the Methods section, including the specific commands, model specifications, and analytical choices made, so that any researcher with access to comparable data could replicate our approach. We believe this level of methodological transparency meets the spirit of PLOS's reproducibility goals under the circumstances described. We hope this explanation is satisfactory and welcome any further discussion. |
| E.3 | Please send a completed 'Competing Interests' statement, including any COIs declared by your co-authors. If you have no competing interests to declare, please state "The authors have declared that no competing interests exist". | Thank you. We added the statement “The authors have declared that no competing interests exist “at the end of the article (line 406). |
| E.4 | In the online submission form you indicate that your data is not available for proprietary reasons and have provided a contact point for accessing this data. Please note that your current contact point is a co-author on this manuscript. According to our Data Policy, the contact point must not be an author on the manuscript and must be an institutional contact, ideally not an individual. Please revise your data statement to a non-author institutional point of contact, such as a data access or ethics committee, and send this to us via return email. Please also include contact information for the third-party organization, and please include the full citation of where the data can be found. | We have revised the Data Availability Statement in accordance with the journal’s Data Policy.  Due to privacy and ethical concerns, the data supporting the findings of this study are not publicly available. For inquiries regarding this, requests should be directed to: [epimail@epicentre.msf.org](mailto:epimail@epicentre.msf.org) |
| E.5 | Please provide separate figure files in .tif or .eps format. | We attached 3 files for figures (.tiff format) |
| **Reviewer** | **Comments** | **Responses to Reviewers** |
| R1 | Have the authors made all data underlying the findings in their manuscript fully available (please refer to the Data Availability Statement at the start of the manuscript PDF file)?  The PLOS Data policy requires authors to make all data underlying the findings described in their manuscript fully available without restriction, with rare exception. The data should be provided as part of the manuscript or its supporting information, or deposited to a public repository. For example, in addition to summary statistics, the data points behind means, medians and variance measures should be available. If there are restrictions on publicly sharing data—e.g. participant privacy or use of data from a third party—those must be specified. | We respectfully request an exemption from full public data sharing on both legal and ethical grounds.  **Data protection considerations** The dataset underlying this study includes individual-level data collected from participants as part of a research collaboration with the local Ministry of Health. Despite efforts to de-identify the data, we cannot guarantee full anonymization within the meaning of the General Data Protection Regulation (GDPR). As a result, these data remain personal data. Making them publicly available without restriction would therefore constitute a breach of GDPR requirements, expose participants to risks of re-identification, and potentially expose our institution (Epicentre) to legal liability.  **Ethical considerations**  In addition, participants consented to the use of their data strictly within the context of this study. They did not consent to unrestricted public sharing of their data. As per the protocol approved by two ethics review boards, Epicentre has a responsibility to ensure that any data sharing is conducted within a controlled framework. This is implemented through a Data Sharing Agreement, which requires recipients to comply with strict confidentiality, data protection, and ethical obligations.  For these reasons, unrestricted public deposition of the dataset would breach both the approved ethical protocol and applicable legal requirements.  We remain committed to transparency and scientific collaboration. Accordingly, the minimal dataset can be made available upon reasonable request to qualified researchers, subject to review and the signature of a Data Sharing Agreement. |
| R.5.1 | Abstract should mention the CAD software and version that was used. | We thank the reviewer for this suggestion. The requested information has been added to the abstract (line 26). |
| R.5.2 | The abstract states that “context specific CAD threshold calibration can improve ACF efficiency…” But this abstract doesn’t really make clear what these “contexts” are that led to adaption. Was it only referral and yield monitoring data? If so, might be better phrased as “monitoring data” or similar. | We thank you the reviewer for this pertinent comment. Indeed, in our study and then in the abstract, “context” refers to multiple operational and programmatic factors used to guide CAD threshold calibration, including observed referral rates, comparison with radiologist sensitivity, screening yield, and broader implementation constraints (such as workflow feasibility and stakeholder feedback). The calibration process was therefore not based solely on monitoring data, but on a combination of real-time programmatic indicators and operational considerations.  To improve clarity, we have revised the final sentence of the abstract to specify the key elements informing calibration. The sentence now reads: “CAD threshold calibration, informed by referral rate targets, screening yield, and operational constraints, can improve ACF efficiency and user confidence.”(line 39-40). |
| R.5.3 | Lines 111-113: would it be possible to present some local data on TB epidemiology here too? Perhaps even trends in pulmonary TB case notification rates? This would help contextualise the setting within which screening was done. | We thank the reviewer for this suggestion. We have added subnational context on tuberculosis epidemiology in the National Capital Region (NCR), which includes Manila, drawing on routine Department of Health National Tuberculosis Program reporting. While city-level notification data for Manila are not systematically available in public reports, NCR-level data consistently show the highest TB burden nationally and reflect persistent transmission in densely populated urban poor settings such as Tondo. (line 110-119) |
| R.5.4 | Lines 201-208: Could the authors please add the crude numbers, as well as the percentages here. | We have added as requested. (line 214) |
| R.5.5 | I thought the qualitative interviews and results were excellent - really informative and practical. | We thank the reviewer for this positive feedback on the qualitative component of the study. We are pleased that these findings were considered informative and practical. A separate manuscript focusing specifically on the feasibility and acceptability aspects of the intervention, including the qualitative results, has been submitted and is currently under review. |
| R.5.6 | In the discussion, one thing that is not discussed is economics - varying thresholds will have cost implications for people offered screening, as well as implication for the programme. It would be worthwhile adding a couple of short sentences about these considerations. | Thank you for this important comment. We have added two sentences to the discussion acknowledging the economic implications of threshold calibration, noting that cost-effectiveness analyses in different programmatic contexts are needed. (line 364-368); We would also like to highlight that we are currently submitting a research proposal to conduct a cost-effectiveness analysis of the full model of care, including threshold calibration aspects. |
